# The Effects of Polystyrene Microplastics and Copper Ion Co-Contamination on the Growth of Rice Seedlings

**DOI:** 10.3390/nano15010017

**Published:** 2024-12-26

**Authors:** Huiyu Jin, Guohe Lin, Mingzi Ma, Lin Wang, Lixiang Liu

**Affiliations:** 1 Miami College, Jinming Campus, Henan University, Kaifeng 475004, China15237847119@163.com (G.L.); 2 Zhoukou Environmental Science and Technology Information Service Center, Zhoukou 466000, China; 3 College of Geographical Sciences, Faculty of Geographical Science and Engineering, Henan University, Zhengzhou 450046, China; 4 State Key Laboratory of Environmental Criteria and Risk Assessment, State Environmental Protection Key Laboratory of Regional Eco-Process and Function Assessment, Chinese Research Academy of Environmental Sciences, Beijing 100012, China

**Keywords:** polystyrene microplastics, heavy metals, Cu^2+^, rice seedlings, peroxidase

## Abstract

Microplastics (MPs) are emerging pollutants of global concern, while heavy metals such as copper ions (Cu^2+^) are longstanding environmental contaminants with well-documented toxicity. This study investigates the independent and combined effects of polystyrene microplastics (PS-MPs) and Cu on the physiological and biochemical responses of rice seedlings (*Oryza sativa* L.), a key staple crop. Hydroponic experiments were conducted under four treatment conditions: control (CK), PS-MPs (50 mg·L^−1^), Cu (20 mg·L^−1^ Cu^2+^), and a combined PS-MPs + Cu treatment. The results showed that PS-MPs had a slight stimulatory effect on root elongation, while Cu exposure mildly inhibited root growth. However, the combined treatment (PS-MPs + Cu) demonstrated no significant synergistic or additive toxicity on growth parameters such as root, stem, and leaf lengths or biomass (fresh and dry weights). Both PS-MPs and Cu significantly reduced peroxidase (POD) activity in root, stem, and leaf, indicating oxidative stress and disrupted antioxidant defenses. Notably, in the combined treatment, PS-MPs mitigated Cu toxicity by adsorbing Cu^2+^ ions, reducing their bioavailability, and limiting Cu accumulation in rice tissues. These findings reveal a complex interaction between MPs and heavy metals in agricultural systems. While PS-MPs can alleviate Cu toxicity by reducing its bioavailability, they also compromise antioxidant activity, potentially affecting plant resilience to stress. This study provides a foundation for understanding the combined effects of MPs and heavy metals, emphasizing the need for further research into their long-term environmental and agronomic impacts.

## 1. Introduction

In recent years, plastics have been widely used in daily life due to their excellent flexibility, low cost, and lightweight properties. Statistics indicate that global plastic production reached approximately 400 million tons in 2022, with China accounting for 32%, Europe 14%, and North America 17% [1]. By 2050, the cumulative global plastic production is expected to reach 33 billion tons [2]. Once plastics enter the natural environment, they gradually degrade into smaller microplastics through weathering, UV radiation, biodegradation, and human activities, demonstrating significant mobility [3]. Microplastics (MPs), defined as plastic particles smaller than 5 mm, are persistent organic pollutants that can remain in soil for extended periods while adsorbing other toxic contaminants [4]. Studies indicate that MPs can be ingested by organisms through the food chain, traverse cellular membranes, cross the blood-brain and placental barriers, and accumulate in tissues and organs, posing severe health risks [5].

When MPs are released into the soil environment, they inevitably interact with crops, subsequently affecting human health through the food chain [6]. Research has shown that MPs inhibit the growth of rice seedlings, disrupt the absorption and accumulation of mineral elements, and reduce biomass [7]. Microplastics, especially polystyrene (PS) microplastics, accumulate in the environment due to their high chemical stability, difficulty in degradation, and wide application, and they have become a focus of ecological risk [8,9]. PS microplastics mainly come from industrial plastic products (such as packaging materials and disposable tableware), building insulation materials, and plastic decomposition particles, which enter water bodies, soil, and the atmosphere through wind erosion, runoff, and domestic sewage [10]. Since PS has high hydrophobicity and surface activity, it can not only adsorb other pollutants in the environment but also accumulate through the food chain, ultimately affecting the ecosystem and human health [11,12]. In environmental media, the long-term existence and ecological hazards of polystyrene microplastics (PS-MPs) are particularly prominent. Additionally, MP exposure induces oxidative stress, damages cellular functions, and keeps rice in a state of sustained oxidation, weakening its mineral transport capacity and affecting nutrient uptake from the soil [13]. The particle size and concentration of MPs play critical roles in their toxicity and transmissibility in rice development. For example, low concentrations of PS-MPs can stimulate the antioxidant system in rice roots, enhancing stress levels and reducing oxidative damage. However, concentrations exceeding 30 mg/L can trigger lipid peroxidation, impairing the antioxidant defense mechanisms in roots [14]. Compared to larger plastic particles, MPs possess higher adsorption capacity due to their larger specific surface area, high porosity, and hydrophobicity. They can adsorb various pollutants in the environment, such as heavy metals, polycyclic aromatic hydrocarbons, plastic additives, pesticides, nanoparticles, and microorganisms [15].

Copper (Cu), as a heavy metal pollutant, is an essential trace element for biological growth and development but exhibits complex toxicity and regulatory effects. The growth of rice is significantly influenced by Cu concentrations. Studies have shown that excessive Cu inhibits rice growth, metabolism, and photosynthesis rates [15], although it can promote rice growth under certain conditions [16]. Furthermore, the concentration of Cu^2+^ in soil has been found to negatively correlate with rice yield. At low concentrations (1 μmol/L), Cu^2+^ has minimal effects on rice seedlings; however, as the concentration increases, Cu’s inhibitory effect on rice seedlings becomes more pronounced [17]. Excessive Cu discharge leads to soil contamination, reduces soil fertility, and ultimately disrupts soil ecosystems [16,17].

Both PS-MPs and Cu, whether individually or in combination, exert varying degrees of impact on soil ecosystems and plant growth. However, existing studies primarily focus on the toxicity effects of MPs’ particle size or their co-occurrence with other heavy metals, with limited research on the combined pollution of Cu and PS-MPs. Rice, as a globally cultivated staple crop, serves as an ideal model plant for studying MPs and heavy metal contamination. Therefore, this study systematically investigates the effects of Cu and PS-MPs, both independently and in combination, on the physiological and biochemical characteristics of rice seedlings, aiming to elucidate their interaction mechanisms. The findings will provide scientific evidence and technical support for soil remediation and the establishment of environmental quality standards for MPs and heavy metal contamination.

## 2. Materials and Methods

### 2.1. Experimental Design

The test plants used in this study were rice seedlings of the variety Guangxingyou 1380, purchased from Hainan Shennong Gene Technology Co., Ltd, Haikou, China. Polystyrene microplastics (PS) with a particle size of 300 mesh were obtained from Kexinda Polymer Materials Co., Ltd., Dongguan, China, and copper sulfate (CuSO_4_·5H_2_O) was of analytical grade. Four treatments were established: a control group (CK), a polystyrene microplastic group (PS), a copper ion group (Cu), and a combined treatment group of polystyrene microplastics and copper ions (PS+Cu). Each treatment included five replicates.

Rice seedlings were grown hydroponically in 500 mL Erlenmeyer flasks, with three uniform and healthy seedlings planted in each flask. Each flask contained 250 mL of culture solution, with different treatments as follows. (1) CK: culture solution; (2) PS group: 50 mg·L^−1^ polystyrene microplastic culture solution; (3) Cu group: 20 mg·L^−1^ Cu^2+^ culture solution; (4) PS+Cu group: 50 mg·L^−1^ polystyrene microplastic solution combined with 20 mg·L^−1^ Cu^2+^ culture solution.

After transplanting the rice seedlings, their roots were fully immersed in the culture solution, and the flask mouths were sealed with aluminum foil. The prepared flasks were placed in an environmental pollution simulation incubator (BRS-WHM-2000G, Ningbo Pulante Instrument Co., Ltd., Ningbo, China) at a constant temperature of 25 °C with an 8 h photoperiod per day for one week. To maintain the stability of the culture solution concentrations, water consumed during seedling growth was replenished daily at noon using a micropipette.

On the 7th day, the following parameters were measured for the rice seedlings in each flask: root length, stem length, fresh weight, dry weight, peroxidase (POD) activity, and copper content.

### 2.2. Preparation of Solutions

To prepare the iron salt solution, 3.73 g of EDTA-Na_2_ and 2.78 g of FeSO_4_·4H_2_O were weighed accurately. A total of 1 L of deionized water was boiled and cooled to 70 °C. A volume of 300 mL of the cooled water was poured into a beaker containing EDTA-Na_2_, followed by stirring until completely dissolved. Another 200 mL of deionized water was cooled and added to a beaker containing FeSO_4_·4H_2_O and stirred until dissolved. The FeSO_4_ solution was then slowly added to the EDTA-Na_2_ solution under continuous stirring. The 500 mL iron salt solution was transferred to a brown glass bottle and stored at low temperature in the dark.

Hoagland nutrient solution was prepared by accurately weighing the following components [18]: 1.18 g Ca(NO_3_)_2_·4H_2_O, 0.51 g KNO_3_, 0.49 g MgSO_4_·7H_2_O, 0.14 g KH_2_PO_4_, 2.86 mg H_3_BO_3_, 1.81 mg MnCl_2_·4H_2_O, 0.22 mg ZnSO_4_·7H_2_O, 0.08 mg CuSO_4_·5H_2_O, and 0.02 mg H_2_FMnO_4_·H_2_O. Each component was dissolved in a beaker in a small volume of distilled water. Before setting the volume, 2 mL of FeEDTA solution was added. The solution was then diluted to a final volume of 1 L with distilled water.

To prepare the Cu^2+^ solution, 0.0786 g of CuSO_4_·5H_2_O was weighed and dissolved in an appropriate volume of the nutrient solution. The solution was stirred using a glass rod and diluted to a final volume of 1 L using a volumetric flask, yielding a 20 mg·L^−1^ Cu^2+^ solution.

To prepare the PS-MPs suspension, 0.0500 g of PS microplastic powder was added to the prepared nutrient solution and diluted to a final volume of 1 L. The suspension was sonicated for 30 min using a 400 W, 20.5 kHz ultrasonic homogenizer to ensure uniform dispersion, resulting in a 50 mg·L^−1^ microplastic suspension.

For preparation of PS and Cu treatment Solution, 0.0500 g of PS microplastic powder was added to a beaker with an appropriate volume of nutrient solution and sonicated in a water bath for 30 min using an ultrasonic homogenizer. Separately, 0.0786 g of CuSO_4_·5H_2_O was weighed and dissolved in the nutrient solution, then diluted to 1 L to prepare the PS microplastic-Cu composite solution.

### 2.3. Measurement Methods

The lengths of root, stem, and leaf were measured using a standard ruler. To determine the fresh weight, rice seedlings were first rinsed with distilled water to remove any debris. Excess water was gently removed by air drying. The fresh weight was then measured using an analytical balance. For dry weight determination, the seedlings were placed into labeled envelopes and dried in an oven at 80 °C until reaching a constant weight.

The activity of peroxidase (POD) was measured using a commercial antioxidant enzyme assay kit (POD kit, Solarbio, Beijing, China). Following the kit’s protocol, approximately 0.10 g of tissue was weighed and homogenized in 1 mL of extraction buffer in an ice bath. The homogenate was centrifuged at 8000× *g* for 10 min at 4 °C using a high-speed refrigerated microcentrifuge (TGL-16S, Shuke Instruments, Chengdu, China). The supernatant was collected and kept on ice for further analysis.

The POD activity assay was performed using a spectrophotometer (721G, Shanghai INESA Scientific Instrument, Shanghai, China) at a wavelength of 470 nm [19]. The reaction mixture was prepared in a 1 mL glass cuvette by sequentially adding 15 μL of sample, 270 μL of distilled water, 520 μL of Reagent 1, 130 μL of pretreated Reagent 2, and 135 μL of Reagent 3. The mixture was immediately stirred to ensure homogeneity, and the reaction was timed. The absorbance values were recorded at 30 s (A1) and 90 s (A2). The change in absorbance (∆A) was calculated as follows:

ΔA = A2 − A1(1)

The POD activity was then calculated using the following formula:

POD Activity (U/g) = 7133 × ΔA ÷ W (2)

where W is the sample weight (g).

The rice seedlings were placed in an oven for further drying until a constant weight was achieved. After drying, the samples were ground into a fine powder and stored in sealed bags for subsequent analysis. Briefly, plant samples were first dried to a constant weight and then finely ground. Approximately 0.5000 g of the powdered sample was digested with 8 mL of concentrated HNO_3_ until the solution became clear. The digested sample was filtered and diluted with deionized water to 50 mL. The Cu content in rice seedlings was determined using an atomic absorption spectrophotometer (AAS).

### 2.4. Data Analysis

Data were organized and plotted using Excel and Origin 2024, and one-way analysis of variance (ANOVA) was performed using SPSS 27.0 software.

## 3. Results and Discussion

### 3.1. Effects of PS-MPs and Cu^2+^ on the Growth of Rice Seedlings

**Figure 1:**
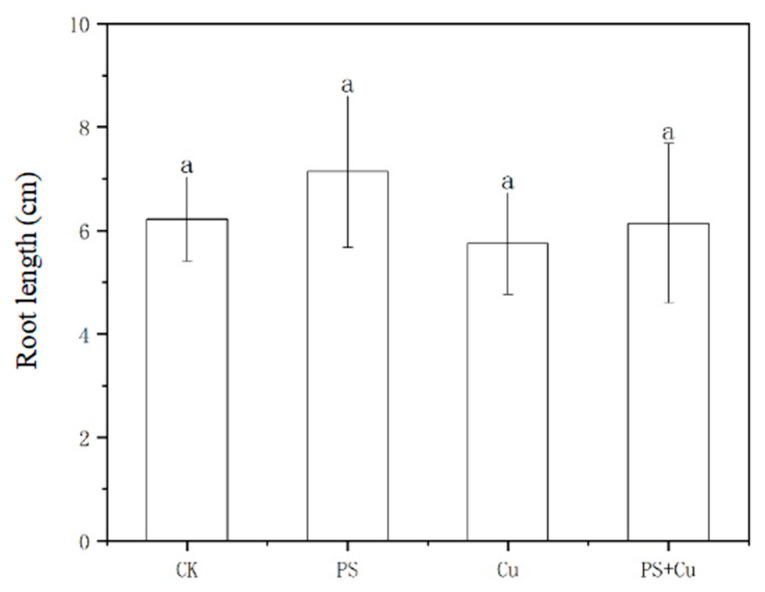
Effect of single and combined treatment of PS-MP and Cu^2+^ on root length of rice seedlings. The letter of a indicates insignificant differences between treatments.

Figure 1 demonstrates the inhibitory effects of PS-MPs and Cu^2+^ on the root length of rice seedlings under single and combined treatments. Exposure to PS-MPs slightly increased root length compared to the control group CK, suggesting minimal physical or chemical interference. This might indicate that at the given concentration, PS-MPs had no significant toxic effect or possibly created a mild priming effect for root elongation. The single treatment of Cu resulted in a slight reduction in root length compared to CK. The observed impact highlights the toxicity of Cu^2+^, potentially through oxidative stress and disruption of cellular functions. The combined treatment of PS+Cu showed a similar root length to CK, with no significant synergistic or additive toxic effects observed. This contrasts with some studies reporting heightened toxicity under combined stress, suggesting either limited adsorption of Cu^2+^ by PS-MPs under experimental conditions or other mitigating factors. Contrary to prior findings, such as those by Zhou et al. [20], which demonstrated that PS-MPs can enhance the toxicity of heavy metals by adsorbing them and increasing their bioavailability, Figure 1 indicates no significant synergistic effects. The observed differences could stem from variations in experimental design, such as PS-MP concentration, Cu^2+^ availability, or differences in plant species and growth conditions. Some studies also suggest that the surface characteristics of PS-MPs can vary, influencing their ability to adsorb heavy metals and their overall interaction with plant roots.

The proposed mechanisms for the observed effects include minimal adsorption and bioavailability, oxidative stress, and the physical impact of PS-MPs. It is possible that PS-MP particles did not effectively adsorb Cu^2+^ under the experimental conditions, thereby limiting the enhancement of Cu toxicity. Additionally, the rice root system’s ability to regulate Cu^2+^ uptake may have mitigated any potential toxic effects of the combined treatment. Regarding oxidative stress, Cu alone might have induced moderate levels, while the presence of PS-MPs could have influenced the plant’s antioxidant response system, potentially offsetting some of the toxicity.

**Figure 2:**
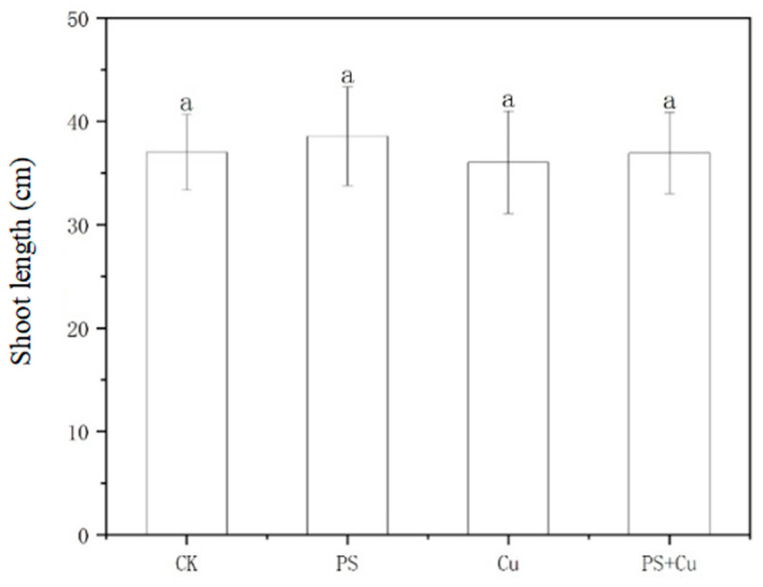
Effect of single and combined treatment of PS-MP and Cu^2+^ on shoot length of rice seedlings. The letter of a indicates insignificant differences between treatments.

For Figure 2, the PS-MP treatment showed no significant deviation in stem and leaf length compared to the control, indicating that PS-MPs alone do not negatively affect the aerial parts of rice seedlings. Similar to PS-MPs, the Cu treatment showed no notable reduction in stem and leaf length compared to the control. This suggests that Cu^2+^ toxicity under these conditions might not be severe enough to impact stem and leaf growth. Combined treatment of PS+Cu also did not significantly affect the length of the stem and leaf, showing results comparable to those of the control. This indicates that there is no apparent synergistic or additive effect of the combined treatment on the aerial growth of rice seedlings. The results suggest that PS-MPs and Cu^2+^, either individually or in combination, have a limited impact on the shoot growth of rice seedlings under the experimental conditions. This finding contrasts with studies, such as those by Zhou et al. [20] and Liu et al. [21], which reported that combined stressors, including microplastics and heavy metals, often impair plant development due to resource allocation to root detoxification processes. The lack of significant effects on shoot growth here might be due to the plant’s efficient physiological and biochemical defense mechanisms in redistributing energy to sustain aerial growth, even under stress.

The toxicity of Cu^2+^ may have been restricted to the root zone, preventing the translocation of Cu^2+^ to the shoot, thereby preserving their growth. The relatively larger biomass of shoots compared to roots might dilute the toxic effects of Cu^2+^, resulting in minimal visible impacts on their growth. PS-MPs may have adsorbed some Cu^2+^ in the growth medium, reducing their availability for uptake and thus alleviating potential toxicity. The rice seedlings might have activated stress response pathways, such as antioxidant systems and ion compartmentalization, to maintain normal growth in shoots despite the presence of PS-MPs and Cu.

### 3.2. Effects of PS-MPs and Cu^2+^ on the Biomass of Rice Seedlings

**Figure 3:**
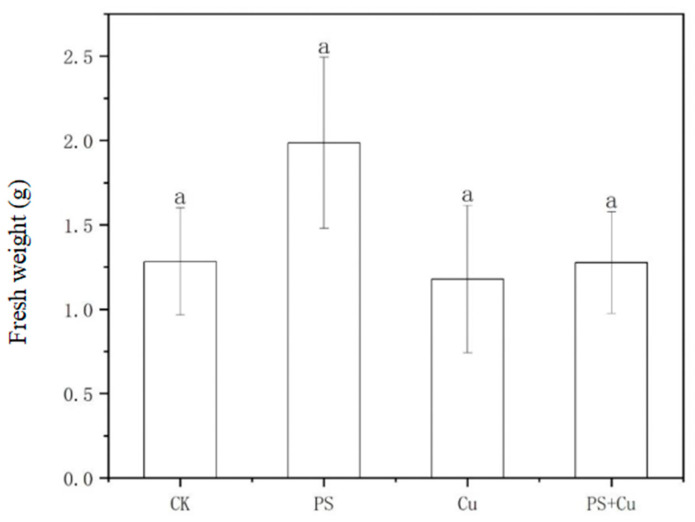
Effect of single and combined treatment of PS-MP and Cu^2+^ on fresh weight of rice seedlings. The letter of a indicates insignificant differences between treatments.

**Figure 4:**
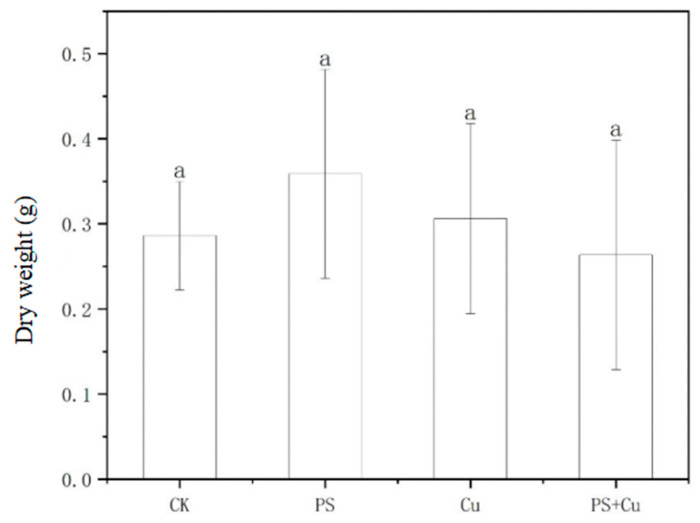
Effect of single and combined treatment of PS-MP and Cu^2+^ on dry weight of rice seedlings. The letter of a indicates insignificant differences between treatments.

Figure 3 and Figure 4 indicate the effects of PS-MPs and Cu, both individually and in combination, on the fresh weight and dry weight of rice seedlings. PS and Cu treatments, either individually or combined, did not have a significant effect on the fresh or dry weight of the rice seedlings. In terms of fresh weight, the PS treatment group showed a slightly higher trend compared to the CK group, but the difference was not statistically significant. This could be attributed to the improvement of soil conditions by low concentrations of microplastics, such as enhanced soil aeration and water retention, which may have promoted rice growth [15,22]. The fresh weight of the Cu treatment and PS+Cu combined treatment groups did not exhibit significant changes, possibly due to the low copper concentration used in the experiment, which did not induce a toxic effect on rice growth. Furthermore, the combined treatment of PS and Cu did not show significant synergistic or antagonistic effects, potentially because PS reduced the bioavailability of Cu^2+^ through adsorption, thereby mitigating its potential toxic effects.

For dry weight, the differences between the treatment groups were also not significant. The dry weight of the PS treatment group was slightly higher than that of the control group, which may indicate a potential stimulatory effect of microplastics on the accumulation of dry matter in rice at suitable concentrations. However, the relatively large variation among treatment groups suggests high data variability, possibly due to individual differences among plants or inconsistencies in experimental conditions [9]. The dry weight of the Cu treatment and PS+Cu combined treatment groups did not show a significant decrease, further supporting the notion that low concentrations of Cu^2+^ have minimal toxic effects on rice seedlings [22,23].

### 3.3. Effects of PS-MPs and Cu^2+^ on Peroxidase Activity in Rice Seedlings

**Figure 5:**
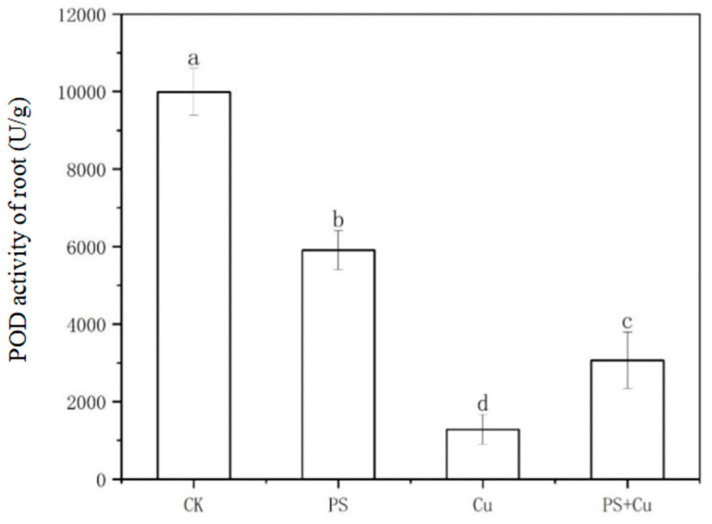
Effect of single and combined treatment of PS-MP and Cu^2+^ on root POD activity of rice seedlings. Different letters a, b, c and d represent significant difference between different treatments (at *p* ≤ 0.05) whereas, same letters indicate insignificant differences between treatments.

**Figure 6:**
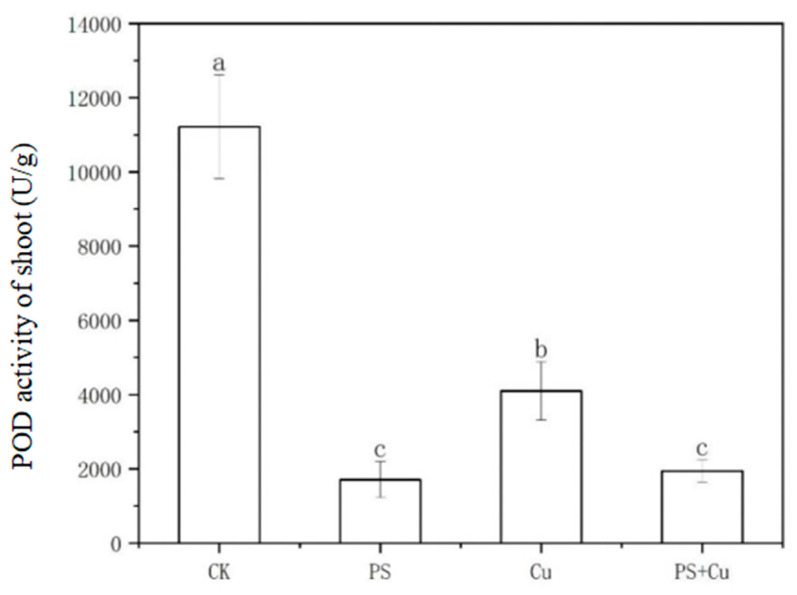
Effect of single and combined treatment of PS-MP and Cu^2+^ on shoot POD activity of rice seedlings. Different letters a, b and c represent significant difference between different treatments (at *p* ≤ 0.05) whereas, same letters indicate insignificant differences between treatments.

Figure 5 and Figure 6 show the effects of PS and Cu^2+^, both individually and in combination, on the peroxidase (POD) activity of rice seedlings. Root POD activity (Figure 5) was highest in the control group CK, significantly higher than in all other treatment groups, indicating that plants maintain a high level of antioxidant enzyme activity under normal growth conditions to combat reactive oxygen species (ROS) in the environment. PS treatment significantly reduced root POD activity, likely due to the disruption of rhizosphere material exchange by microplastics, which induced mild stress and weakened the antioxidant capacity [13,14]. Cu treatment also caused a significant decrease in root POD activity, possibly because copper ions bind to enzyme active sites, thereby inhibiting normal enzyme function [23]. In the combined treatment group (PS+Cu), root POD activity was slightly higher than in the Cu group, suggesting that PS mitigated some of the toxicity of Cu by adsorbing Cu^2+^, though it did not fully restore POD activity [15].

Stem and leaf POD activity (Figure 6) followed a similar pattern, with the CK group showing the highest activity, significantly higher than all other treatments, highlighting the critical role of POD activity in maintaining redox balance under non-stress conditions. PS treatment significantly reduced stem and leaf POD activity, likely due to the inhibition of water and nutrient transport from the roots to the shoots by microplastics, indirectly impairing the antioxidant system [13]. Cu treatment induced significant oxidative stress, resulting in higher stem and leaf POD activity compared to the PS and PS+Cu groups, though it remained lower than in the CK group. This suggests that plants activated POD to alleviate copper toxicity [23]. The stem and leaf POD activity in the PS+Cu group was similar to that in the PS group but significantly lower than in the CK and Cu groups, indicating that while PS adsorption of copper ions may have alleviated some copper toxicity, the suppressive effects of PS on the antioxidant system dominated in the stem and leaf [15,24]. In summary, both PS and Cu treatments, individually and in combination, had significant effects on the POD activity in the roots and shoots of rice seedlings, likely involving varying degrees of interference with the antioxidant system and complex interactive mechanisms.

### 3.4. Effects of PS-MPs and Cu^2+^ on Cu Content in Rice Seedlings

**Figure 7:**
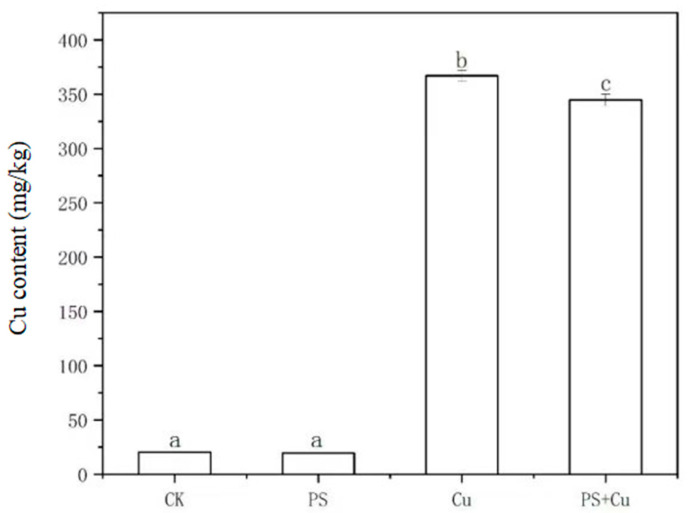
Effect of single and combined treatment of PS-MP and Cu^2+^ on Cu content of rice seedlings. Different letters a, b and c represent significant difference between different treatments (at *p* ≤ 0.05) whereas, same letters indicate insignificant differences between treatments.

Figure 7 illustrates the effects of PS-MPs and Cu treatments, both individually and in combination, on the Cu content in rice seedlings. The results indicate that the Cu content in the CK and PS groups was the lowest, with no significant differences between them, suggesting that PS alone did not significantly alter Cu accumulation in the absence of exogenous Cu. The Cu^2+^ content in the Cu treatment group increased significantly, demonstrating that exogenous Cu markedly enhanced the uptake and accumulation of Cu in rice seedlings, consistent with typical absorption patterns of plants under high concentrations of heavy metals [23]. Furthermore, the Cu^2+^ content in the PS+Cu treatment group was significantly lower than in the Cu treatment group but higher than in the CK and PS groups, indicating that PS reduced Cu^2+^ bioavailability through adsorption, thereby partially decreasing Cu uptake by rice plants. This detoxification effect is consistent with the findings of previous studies [15,24].

The effects of microplastics on Cu accumulation may involve multiple mechanisms. On the one hand, PS directly reduces the bioavailability of Cu^2+^ in the rhizosphere through physical adsorption, limiting Cu uptake by plants. On the other hand, PS may indirectly alter rhizosphere conditions (e.g., pH or the composition of root exudates), further influencing Cu bioavailability [13,14]. Additionally, the absence of significant changes in Cu content under PS treatment alone suggests that PS adsorption has a limited impact. In conclusion, the combined effects of PS and Cu treatments on Cu uptake by rice seedlings indicate that the presence of microplastics can partially mitigate Cu toxicity to plants, though further studies are needed to elucidate the specific interaction mechanisms and their long-term implications.

## 4. Conclusions

This study aimed to investigate the effects of PS-MPs and Cu^2+^, both individually and in combination, on the growth, biomass, antioxidant activity, and Cu uptake of rice seedlings. The main conclusions are as follows:

(1) PS-MPs exhibited a slight stimulatory effect on root growth, while Cu^2+^ alone slightly inhibited root elongation. The combined treatment showed no significant synergistic or additive toxicity. Similarly, shoot lengths, as well as fresh and dry weights, were not significantly affected under any treatment, indicating limited impact of low-concentration PS-MPs and Cu on overall rice growth;
(2) Both PS-MPs and Cu^2+^ significantly reduced POD activity in roots and shoots. While the combined treatment partially alleviated Cu toxicity, the suppressive effects of PS-MPs on the antioxidant system were predominant;
(3) PS-MPs reduced Cu^2+^ bioavailability through adsorption, resulting in decreased Cu accumulation in rice seedlings under the combined treatment and mitigating Cu toxicity.

In conclusion, PS-MPs can alleviate heavy metal toxicity by reducing metal bioavailability. Future research will explore dose-dependent effects to assess the potential thresholds for toxicity and their impact on plant growth. Additionally, incorporating a broader range of biomarkers, the long-term interactive mechanisms between microplastics and heavy metals would provide a more comprehensive understanding of the plant’s stress response.

## Data Availability

All related data are provided within the manuscript.
